# Supplementary material for: Observation of flat band, Dirac nodal lines and topological surface states in Kagome superconductor CsTi3Bi5
Source: Nat Commun. 2023 Jul 10;14:4089. doi: 10.1038/s41467-023-39620-0 (PMC10333313; doi:10.1038/s41467-023-39620-0)
Supplement: Supplementary file 1 — Supplementary Information [file 41467_2023_39620_MOESM1_ESM.pdf]

Supplementary Information:

Observation of Flat Band, Dirac Nodal Lines and Topological Surface States in Kagome Superconductor $CsTi_3Bi_5$

Jiangang Yang[1,2,♯], Xinwei Yi[2,♯], Zhen Zhao[1,2,♯], Yuyang Xie[1,2,♯], Taimin Miao[1,2], Hailan Luo[1,2], Hao Chen[1,2], Bo Liang[1,2], Wenpei Zhu[1,2], Yuhan Ye[1,2], Jing-Yang You[3], Bo Gu[2,4,5], Shenjin Zhang[6], Fengfeng Zhang[6], Feng Yang[6], Zhimin Wang[6], Qinjun Peng[6], Hanqing Mao[1,2,7], Guodong Liu[1,2,7], Zuyan Xu[6], Hui Chen[1,2,4], Haitao Yang[1,2,4], Gang Su[2,4,5,*], Hongjun Gao[1,2,4,*], Lin Zhao[1,2,7,*] and X. J. Zhou[1,2,7,*]

[1]*Beijing National Laboratory for Condensed Matter Physics, Institute of Physics, Chinese Academy of Sciences, Beijing 100190, China.*

[2]*School of Physical Sciences, University of Chinese Academy of Sciences, Beijing 100049, China.*

[3]*Department of Physics, Faculty of Science, National University of Singapore, Singapore 117551, Singapore.*

[4]*CAS Center for Excellence in Topological Quantum Computation, University of Chinese Academy of Sciences, Beijing 100190, China*

[5]*Kavli Institute of Theoretical Sciences, University of Chinese Academy of Sciences, Beijing, 100190, China.*

[6]*Technical Institute of Physics and Chemistry, Chinese Academy of Sciences, Beijing 100190, China.*

[7]*Songshan Lake Materials Laboratory, Dongguan, Guangdong 523808, China.*

[♯]*These authors contribute equally to the present work.*

[*]*Corresponding author: hjgao@iphy.ac.cn, gsu@ucas.ac.cn, lzhao@iphy.ac.cn and XJZhou@iphy.ac.cn*

Supplementary Figure 1. Original Fermi surface mappings of $CsTi_3Bi_5$ over three momentum regions.

Supplementary Figure 2. Origin of the flat band in $CsTi_3Bi_5$.

Supplementary Figure 3. The buildup of the spectral weight in the binding energy range of 250$\sim$500 meV across the entire Brillouin zone.

Supplementary Figure 4. Comparison between the measured band structures and the calculated kz-integrated band structures for $CsTi_3Bi_5$.

Supplementary Figure 5. Calculated distribution of the NL1 and NL2 Dirac nodal lines in three-dimensional Brillouin zone.

Supplementary Figure 6. Nontrivial $\mathbb{Z}_2$ invariant of bands near the Fermi level.

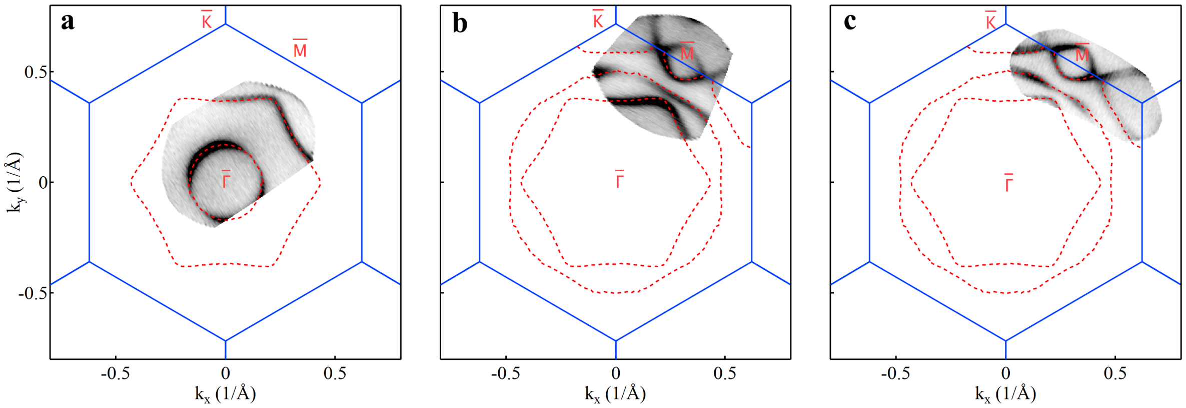

Supplementary Figure 1: Original Fermi surface mappings of $CsTi_3Bi_5$ over three momentum regions. The Fermi surface in Fig. 1 is obtained from these results by considering six-fold symmetry.

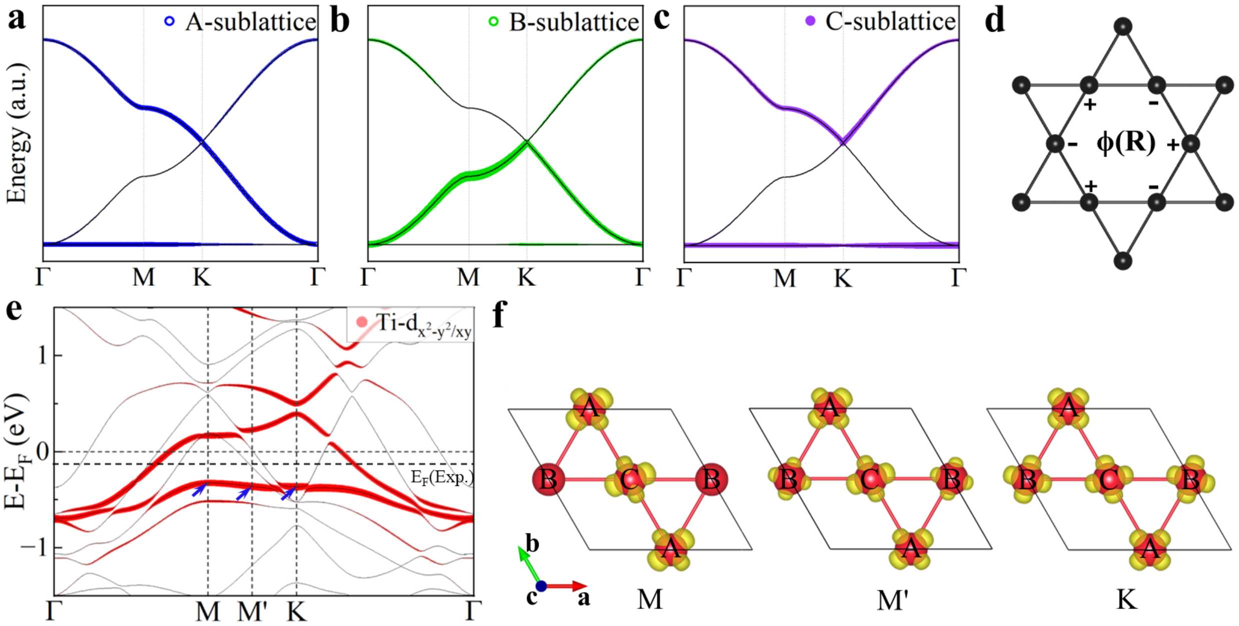

Supplementary Figure 2: Origin of the flat band in $CsTi_3Bi_5$. a-c Electronic band structures of the tight-binding model of kagome lattice weighted by projected composition of three sublattices A, B, and C, respectively. **d** The localized eigenstate of the flat band in (a-c), where "+" and "-" represent the sign of wavefunctions of different sites. **e** DFT electronic band structures of $CsTi_3Bi_5$ with considering SOC. **f** Charge density distribution of the flat band states at the M, M', and K respectively. The charge density distribution is drawn with an isosurface of about 0.004 $e/Bohr^3$. These three electronic states are marked by blue arrows in (e).

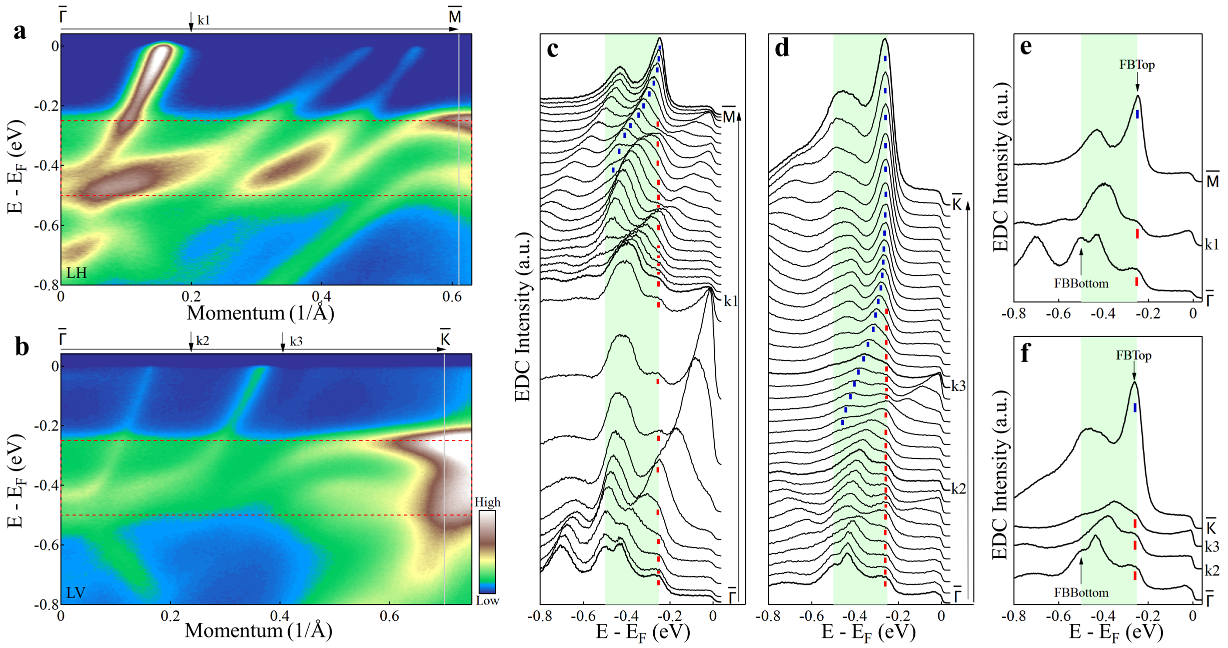

Supplementary Figure 3: The buildup of the spectral weight in the binding energy range of 250∼500 meV across the entire Brillouin zone. a,b Detailed band structures measured along the $\bar{\Gamma}$-$\bar{M}$ and $\bar{\Gamma}$-$\bar{K}$ high symmetry directions, respectively. The spectral weight buildup regions are marked by the dashed red frames. **c,d** The corresponding EDCs from **a** and **b**, respectively. For clarity, the EDCs are offset along the vertical axis. The blue ticks mark the energy position of kagome lattice-derived flat band. The red ticks mark the cutoff energy position of the spectral weight buildup at the binding energy of ∼250 meV. The shaded regions correspond to the spectral weight buildup regions in **a** and **b**. **e,f** Representative EDCs selected from **c** and **d**, respectively, in order to highlight the flat band energy positions and the cutoff position of the spectral weight buildup. The flat band top (FBTop) lies at ∼250 meV as marked by the peak position of the EDCs at $\bar{M}$ and $\bar{K}$. The flat band bottom (FBBottom) lies at ∼500 meV as marked by the peak position of the EDCs at $\bar{\Gamma}$. The flat band top and bottom energy positions coincide with the top and bottom cutoff positions of the extra spectral weight buildup, indicating that they are closely related to each other.

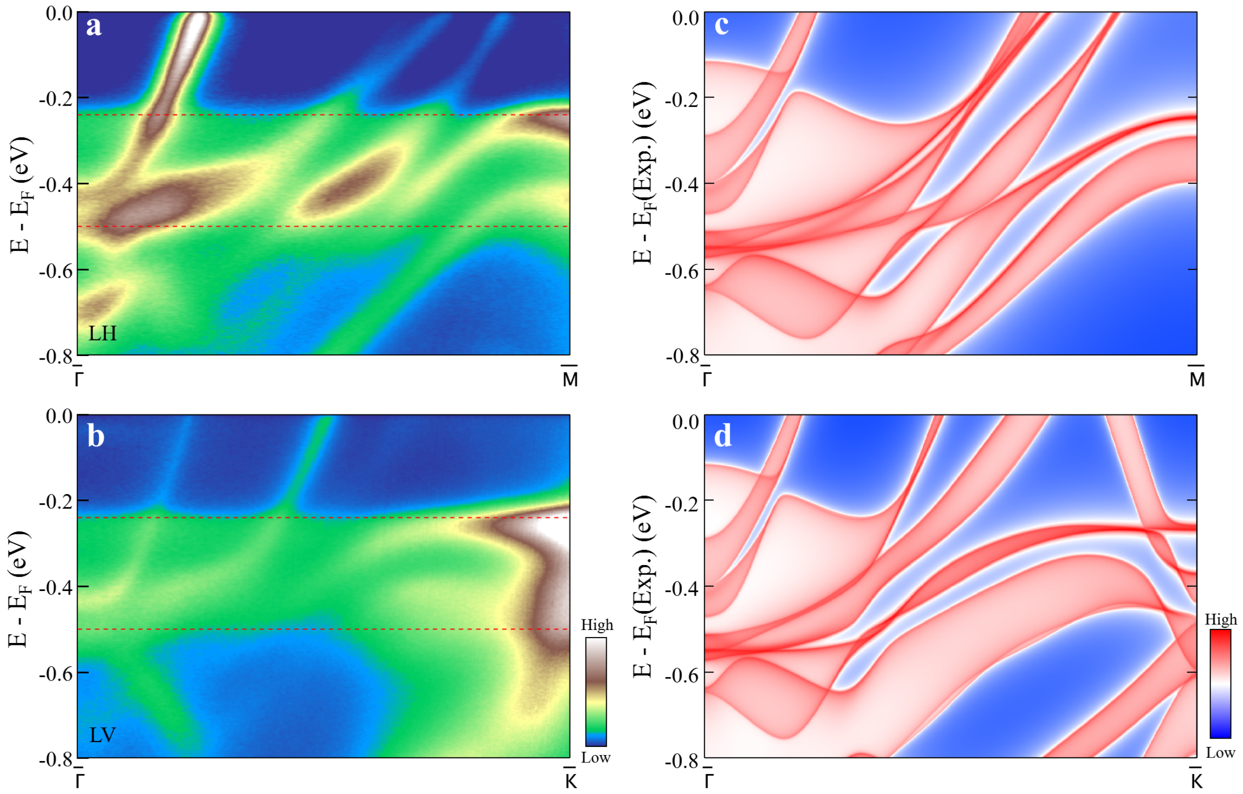

Supplementary Figure 4: **Comparison between the measured band structures and the calculated kz-integrated band structures for $CsTi_3Bi_5$. a,b** Detailed band structures measured along the $\bar{\Gamma}$-$\bar{M}$ and $\bar{\Gamma}$-$\bar{K}$ high symmetry directions, respectively. The spectral weight buildup regions between -0.25∼-0.50 eV are marked by the dashed red frames. **c,d** Calculated kz-integrated band structures along the $\bar{\Gamma}$-$\bar{M}$ and$\bar{\Gamma}$-$\bar{K}$ high symmetry directions.

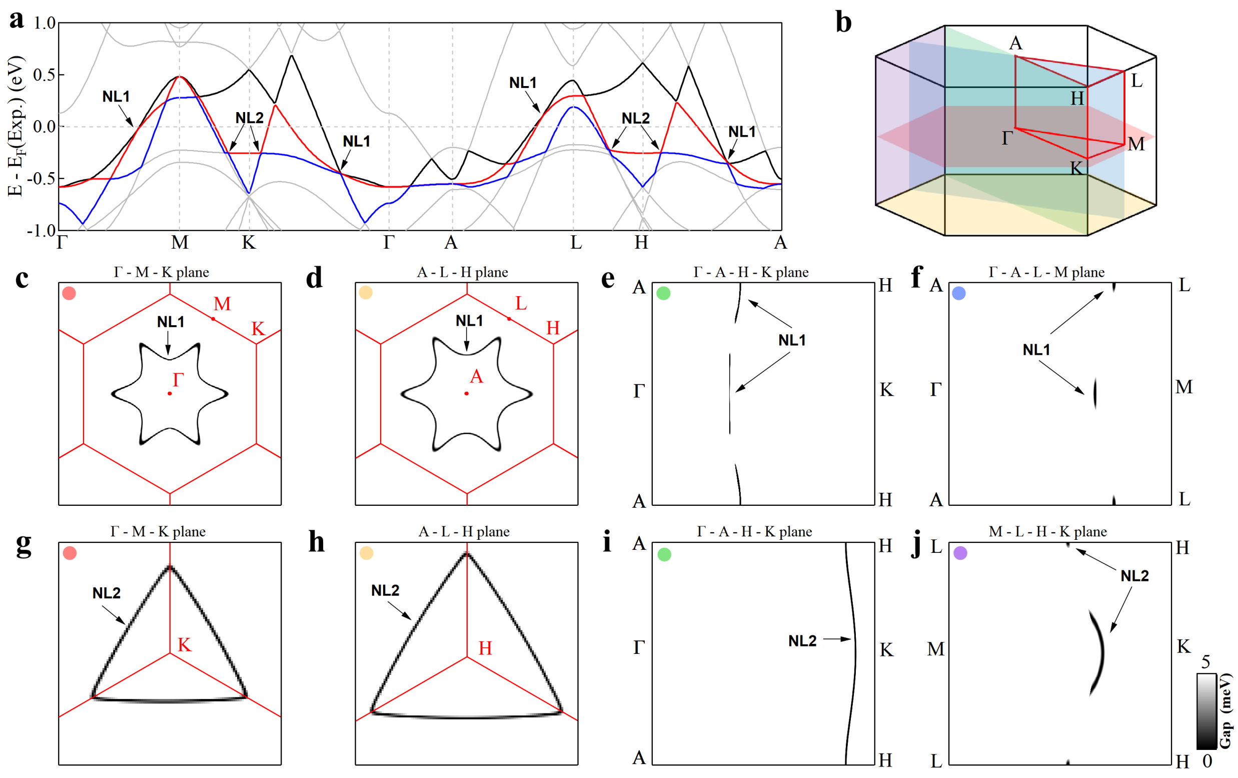

Supplementary Figure 5: Calculated distribution of the NL1 and NL2 Dirac nodal lines in three-dimensional Brillouin zone. **a** Electronic band structures without SOC for $CsTi_3Bi_5$. The β, γ, and δ bands are drawn with black, red and blue colors, respectively. Two sets of Dirac nodal points (NL1 and NL2) are marked by arrows. **b** Three-dimensional Brillouin zone of $CsTi_3Bi_5$ with high symmetry points and high symmetry planes marked. **c,d** The formation of the NL1 Dirac nodal loops in the k_z=0 and k_z=1 planes, respectively. **e,f** Discrete NL1 Dirac nodal lines along kz direction in the Γ-A-H-K plane (e) and the Γ-A-L-M plane (f). **g,h** The formation of the NL2 Dirac nodal loops in the k_z=0 and k_z=1 planes, respectively. **i** Continuous NL2 Dirac nodal lines along k_z direction in the Γ-A-H-K plane. **j** Discrete NL2 Dirac nodal lines along k_z direction in the M-L-H-K plane. The energy gap between the corresponding bands is represented by the bottom-right colar scale. The top-left color legends in **c-j** correspond to the colors of the planes that are drawn in **b**.

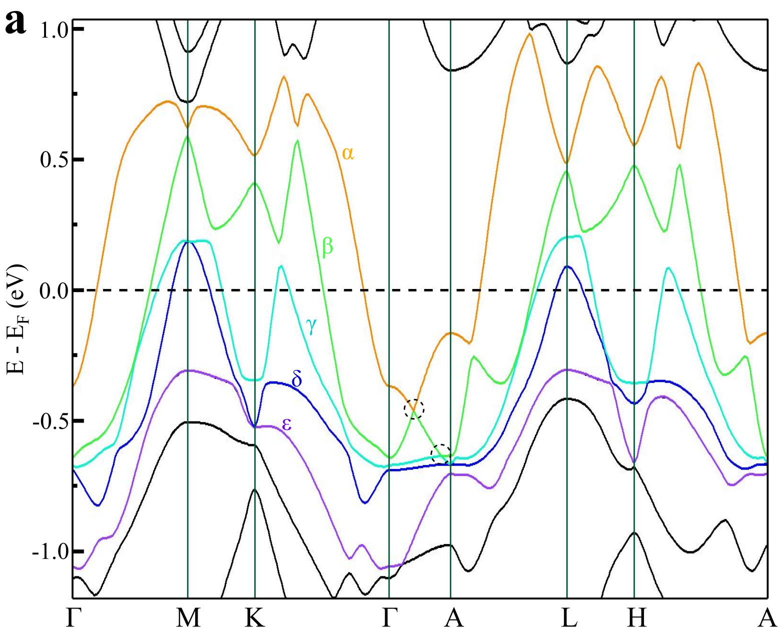

b

Band	Product of Parity				Z_2
	Γ	M	A	L	
δ	-	-	-	+	1
ε	-	+	+	+	1

Supplementary Figure 6: **Nontrivial $\mathbb{Z}_2$ invariant of bands near the Fermi level. a** Calculated electronic bands with SOC for $CsTi_3Bi_5$. The α, β, γ, δ and ϵ bands are drawn with orange, green, light blue, blue, and purple colors, respectively. Band crossings along the Γ-A path between the γ and β bands, as well as between the β and α bands are marked by dashed circles. Continuous band gaps throughout the whole Brillouin zone exist between the ϵ and δ bands, as well as between the δ and γ bands. **b** Products of parity for the four TRIM points and $\mathbb{Z}_2$ invariant for the δ and ϵ bands.

Supplementary Note 1. Destructive interference origin of flat band in $CsTi_3Bi_5$

Destructive interference is a common feature of most flat band models in condensed matter physics, including flat band kagome lattice. This destructive interference mechanism can be well explained by a basic nearest-neighbor (NN) tight-binding model. There are three inequivalent sublattices, namely, A, B and C in the unit cell of the kagome lattice. One single orbital on each sublattice is considered. Taking into account only NN hopping of the three sublattices, a three-band momentum space Hamiltonian can be written as:

$$h(k) = \begin{pmatrix} 0 & 2t\cos(\frac{k\cdot a_1}{2}) & 2t\cos(\frac{k\cdot a_2}{2}) \\ 2t\cos(\frac{k\cdot a_1}{2}) & 0 & 2t\cos(\frac{k\cdot(a_1+a_2)}{2}) \\ 2t\cos(\frac{k\cdot a_2}{2}) & 2t\cos(\frac{k\cdot(a_1+a_2)}{2}) & 0 \end{pmatrix} \quad (1)$$

where t, a_1, a_2 and k represent the NN hopping parameter, lattice vectors and wave vector of reciprocal space, respectively. The eigenvalues of this matrix are $E_1 = -2t$ and $E_{2,3} = t[1 \pm \sqrt{4(cos^2(\frac{k\cdot a_1}{2}) + cos^2(\frac{k\cdot a_2}{2}) + (cos^2(\frac{k\cdot a_1+a_2}{2}))}]$ as plotted in Supplementary Fig. 2a-2c. The Bloch eigenstates of the flat band $E_1 = -2t$ can be simply written as:

$$\phi(k) = \sin(\frac{k\cdot(a_1+a_2)}{2})c^{\dagger}_{k,A} - \sin(\frac{k\cdot a_2}{2})c^{\dagger}_{k,B} + \sin(\frac{k\cdot a_1}{2})c^{\dagger}_{k,C}. \quad (2)$$

Now let us calculate the wannier function of the flat band by Fourier transformation of the Bloch eigenstates:

$$\phi(R) = N\int_{BZ} dk e^{-ik\cdot R}\phi(k) = \frac{1}{\sqrt{6}}\sum_{a=1}^{6}(-1)^a c^{\dagger}_a. \quad (3)$$

where N is a normalization constant and a runs over the six vertices of a hexagon centered at the chosen R. As shown in Supplementary Fig. 2d, $\phi(R)$ takes the form of a localized hexagonal plaquette[1]. The wavefunctions keep the same amplitude but change signs sequentially at six vertices of the hexagon. This causes destructive interference of the wave functions at the six vertices of the hexagram. There is no possibility of an electron hopping from the hexagon to six vertices of the hexagram and hence traveling to the outside lattice. According to Eq. (1) and Eq. (2), the bands in Supplementary Fig. 2a-2c can be colored by weights of the projected composition of three sublattices. We can see that the weights of the three sublattices along high-symmetry paths are different in the flat band and two dispersion bands. For example, the lower van Hove singularity at point M corresponds to

pure sublattice character of B, while the upper van Hove singularity and flat band at the M point show mixed sublattice character of A and C.

Part of the flat band of $CsTi_3Bi_5$ observed by ARPES shows a good agreement with DFT calculations in Figs. 2c and 2d. To confirm that the flat band near -0.25 eV comes from the flat band generated by destructive interference in the kagome tight-binding model, we analyzed the Bloch eigenstates of the flat band of $CsTi_3Bi_5$ at several specific momenta, including M, M' and K points as marked in Supplementary Fig. 2e. Their real space charge densities are plotted in Supplementary Fig. 2f. For different k-points, the ratios of the three sublattice charge densities are quite different. At the M point, the charges are equally distributed near the A and C sublattices, but not near the B sublattice. At the M' point, the charge near A and C sublattices are equivalent, and each of them is much larger than that of the B sublattice. While at the K point, A, B and C sublattices have exactly the same charge density distributions near them. These features are consistent with those of the flat band of the tight-binding model in Supplementary Fig. 2a-2c. These results show that the flat band formed by Ti-$d_{x^2-y^2/xy}$ orbital near -0.25 eV in DFT calculations originated from the destructive interference mechanism of the kagome lattice.

Supplementary References

[1] Liu, Z., Liu, F., Wu, Y.-S. Exotic electronic states in the world of flat bands: From theory to material. *Chin. Phys. B*, 23:077308, 2014.
